# In Vitro Comparison of Polymerization Techniques on the Strength and Hardness of Denture Base Resins

**DOI:** 10.7759/cureus.75132

**Published:** 2024-12-05

**Authors:** Arjun S, Anna S Babu, Krishnapriya V N, Venkitachalam Ramanarayanan, Manju V, Vinod P Nair

**Affiliations:** 1 Prosthodontics and Implantology, Amrita School of Dentistry, Amrita Vishwa Vidyapeetham, Kochi, IND; 2 Public Health Dentistry, Amrita School of Dentistry, Amrita Vishwa Vidyapeetham, Kochi, IND

**Keywords:** acralyn, conventional resins, denture base resins, flexural strength, high-impact resin, impact strength, polymerization cycle

## Abstract

Purpose: Acrylic dentures fabricated using polymethyl methacrylate are subjected to either intraoral fatigue stress or extraoral impact stress, eventually forming microcracks and fractures. This limitation should be overcome by either modification in the acrylic resin material or in polymerization techniques. This study compares the impact strength and flexural strength of high-impact resin to conventional resin in short- and long-heat polymerization settings.

Methodology: The in vitro study design adopted a comparison of two groups of acrylic resin specimens, conventional and high-impact resin groups, with two subgroups, each undergoing short and long curing cycles. Specimens' flexural strength and impact strength were tested using the three-point bending test and Charpy’s impact test (notched), respectively. The comparative analysis between the outcomes of curing cycles for each material and between the materials for each curing cycle was performed using the Mann-Whitney U test.

Results: The results of our study highlighted that the high-impact resin group with short cycles displayed statistically significant values on the flexural and impact strength. A comparison of flexural strength for the conventional resin group in different curing cycles showed that the short cycle had higher flexural strength than the long cycle, which was statistically significant. However, the difference in impact strength between curing cycles in the conventional resin group was not statistically significant.

Conclusion: High-impact denture base resin with short cycles will enhance denture properties with better flexural and impact strength.

## Introduction

Conventional removable dentures have been the patient's preferred choice of dental rehabilitation for the replacement of partial or completely missing teeth due to their affordability. Even though fixed dentures and implant prosthetics have emerged as highly predictable treatment options for edentulous patients, removable dentures always remain a reliable alternative in patients with compromised oral health conditions. It accounts for a non-invasive and reversible treatment option with a reasonable cost and easier hygiene maintenance. The final outcome of the prosthesis depends on good clinical skill along with laboratory expertise and the properties of the material used in processing dentures.

Since the 1940s, acrylic resins have been used in dentistry for various dental procedures [1]. Polymethyl methacrylate (PMMA) has been a prime material among denture-based materials due to its superior physical and mechanical properties [2]. PMMA also provides the qualities of an ideal denture base material, such as good compatibility with oral tissues, stability, longevity, and satisfactory aesthetics. However, a major limitation of acrylic resin is the inferior strength, which leads to fracture of dentures due to impact forces on high masticatory loads. The repetitive force induces the formation of microcracks, which progress to fracture in weak portions of the denture like the midline [3].

Factors like composition, water-powder ratio, and type of polymerization influence the strength of acrylic denture base resins. Different forms of PMMA resins are heat-polymerizable, auto-polymerizing, thermoplastic, light-activated, and microwave-polymerizable. Among these, heat-cured or heat-polymerizable acrylic is still the most widely utilized resin for the fabrication of permanent prostheses as it offers a longer shelf life. In spite of its durability, the material has less impact strength and flexural strength [4].

To overcome these limitations, several research works were carried out by either modifying the composition through the addition of filler particles or polymerization by altering the curing cycle. One of the new concepts in chemical modification was the incorporation of butadiene styrene, a form of rubber, to enhance the impact strength. The PMMA reinforced with rubber is known as high-impact resin [5]. Limited studies are comparing the efficiency of conventional resins with high-impact resins.

In the context of polymerization, the most frequently used, easy, and inexpensive method is conventional hot water bath processing. The main disadvantage of this method is the long processing time. Different curing cycles are proposed in the literature, with variations in time and temperature. These include short cycles, long cycles, and combinations [6]. Moreover, these alterations will prove effective only if the specific mechanical properties like hardness and flexural strength, which improve the longevity of the prosthesis, are enhanced.

Therefore, this study aimed to evaluate and compare the effect of polymerization cycles on specific properties of conventional and high-impact denture base resins. The null hypothesis proposed was that there is no significant difference in the impact strength between conventional heat-cured resin and high-impact resin and that the duration of the curing cycle does not influence the flexural strength of the resins.

This article was previously published as a meeting abstract at the 2024 Federation Dentaire International World Dental Congress on September 15, 2024 [7].

## Materials and methods

Study design

**Figure 1:**
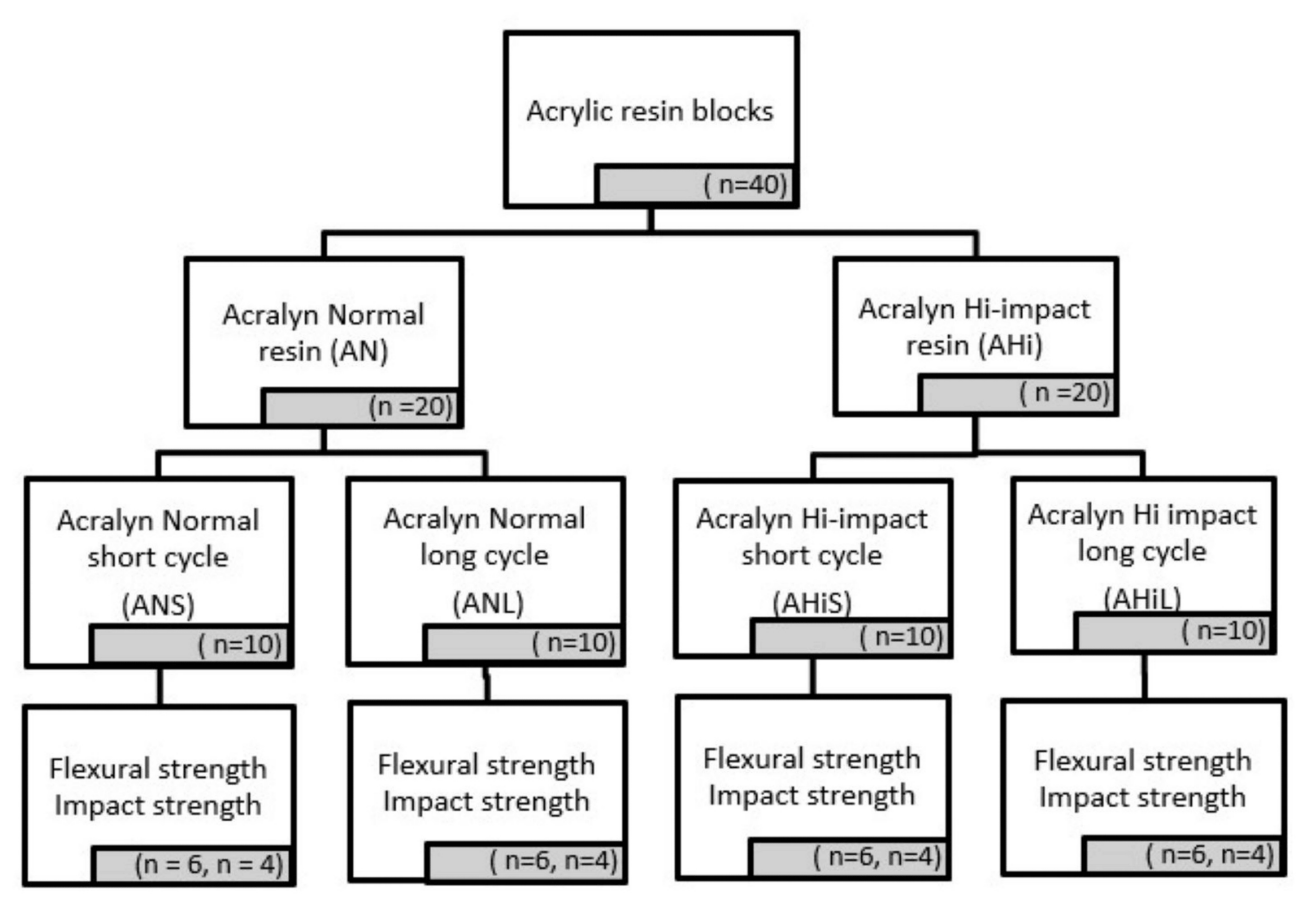
Study flow chart

The study adopted an in vitro design. The two groups of acrylic resins were conventional (Acralyn normal) and high-impact (Acralyn Hi-impact) resin groups. There were two subgroups each for the groups to undergo a short curing cycle (70°C for 90 minutes followed by 100°C for 30 minutes) and long curing cycles (74°C for eight hours), making a total of four subgroups (Figure 1). Thus, the four study groups considered for this study were Acralyn normal undergoing a short cycle, Acralyn normal undergoing a long cycle, Acralyn Hi-impact undergoing a short cycle, and Acralyn Hi-impact undergoing a long cycle.

Materials

**Figure 2:**
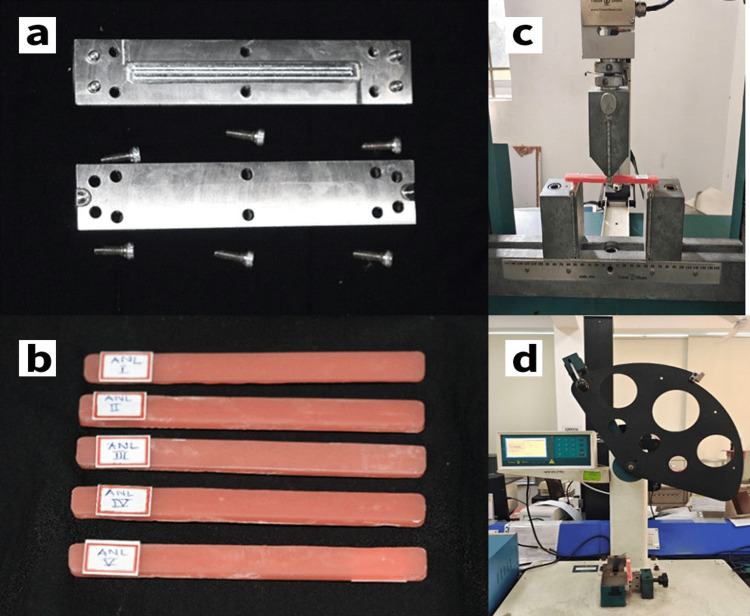
(a) Stainless steel mold; (b) acrylic block specimens; (c) testing of acrylic block for flexural strength in a universal testing machine; (d) testing of acrylic block for impact strength in an impact testing machine

Two types of denture base resins/acrylic resin and two curing protocols of processing were experimented with in this study. The denture base materials used in the study were Asian Acralyn normal and Acralyn Hi-impact resin, polymer, and monomer. Specimens were prepared in a stainless steel mold to get rectangular acrylic blocks of dimension 127 mm × 4 mm × 12 mm (Figure 2a). The heat curing was performed using the conventional water bath (ACRYLIZER C-73A) in the laboratory after setting the temperature and time as per the required protocol. An effect size of 0.90 was considered, resulting in 20 samples in each acrylic resin group (a total of 40 samples). They were further subdivided into 10 samples each for short and long cycles.

Preparation of specimen

All specimens were processed according to the manufacturer’s instructions. The appropriate amount of polymer (powder) and monomer (liquid) were mixed according to the prescribed technique. The mixed material underwent the physical stages of polymerization and was packed into the metallic mold cavity and tightened with screws. This was followed by bench curing for 30 minutes and then placing in a conventional water bath for curing. Two protocols/cycles were adopted for curing with blocks of each group resin: a short cycle and a long cycle [8]. After curing, the specimens were finished, polished, and stored in distilled water (Figure 2b).

Polymerization cycles

Two curing protocols were applied in the study. In the short curing cycle, Acralyn normal specimens and Acralyn Hi-impact specimens will be processed at 70°C for 90 minutes, followed by 100°C for 30 minutes in a conventional water bath. In a long curing cycle, the Acralyn normal specimens and Acralyn Hi-impact specimens will be at 74°C for eight hours in a conventional water bath.

Testing of samples

The flexural strength and impact strength testing of acrylic block specimens were performed at a certified testing center. Forty acrylic blocks were tested, 10 samples per study group. Among these, six samples were used for testing flexural strength, and the rest of the four samples for impact strength.

Three-Point Bending

The flexural strength of acrylic resin was assessed with a universal testing machine (UTM, Model: H50kl, Tinius Olsen). The acrylic blocks were placed in a 50-mm-long support for three-point flexural testing. A vertical load was then applied at the midpoint of the block at a crosshead speed of 5 mm/minute on a load testing machine [9]. The load was applied until failure, and the fracture load was recorded in MPa (Figure 2c).

Charpy’s Impact Test (Notched)

The impact strength was measured at room temperature in a pendulum impact testing machine (Model: IT 504, Tinius Olsen). The device is designed with a pendulum that can apply different weights to the materials to be tested. The specimens were held vertically at one end and struck by the pendulum at the center of the acrylic block tested in the notch area [10]. The scale reading provides the values of Charpy’s impact strength (notched) in J/m (Figure 2d).

Statistical analysis

The analysis was performed using SPSS Statistics version 20.0 (IBM Corp. Released. IBM SPSS Statistics for Windows, Version 20.0. Armonk, NY: IBM Corp.). Flexural strength and impact strength of two denture base resins were estimated and reported as mean ± standard deviation and median (IQR). A comparison of outcomes between the curing cycles for each material and between the materials for each curing cycle was performed using the Mann-Whitney U test. A non-parametric test was used due to the number of samples. A p-value <0.05 was considered statistically significant.

## Results

A total of 40 samples (10 in each group) were analyzed. Descriptive statistics of flexural strength and impact strength between different study groups have been presented in Table 1.

**Table 1:** Descriptive statistics of flexural and impact strength of different study groups SD: standard deviation, IQR: interquartile range

Group	Flexural strength (n=6)		Impact strength (n=4)	
	Mean ± SD	Median (IQR)	Mean ± SD	Median (IQR)
Acralyn normal (short cycle)	52.73 ± 2.68	52.45 (50.40, 54.90)	63.59 ± 5.82	61.19 (60.11, 67.07)
Acralyn normal (long cycle)	46.81 ± 4.89	48.10 (46.20, 48.50)	55.73 ± 5.89	53.90 (51.90, 59.57)
Acralyn Hi-impact (short cycle)	57.08 ± 0.74	56.95 (56.60, 57.80)	167.53 ± 12.68	165.20 (157.98, 177.09)
Acralyn Hi-impact (long cycle)	49.38 ± 1.42	49.15 (48.30, 50.50)	123.39 ± 1.46	123.15 (122.34, 124.46)

A comparison of flexural strength for Acralyn normal material with different curing cycles showed that the short cycle had a higher flexural strength (median: 52.45 MPa) compared to the long cycle (median: 48.10 MPa). This difference was statistically significant (p=0.015). Comparison of flexural strength for Acralyn Hi-impact material with different curing cycles showed that the short cycle had a high flexural strength (median: 56.95 MPa) compared to the long cycle (median: 49.15 MPa), which was statistically significant (p=0.002) (Table 2).

**Table 2:** Comparison of flexural and impact strength between short and long cycles for each material * significant at p<0.05

Group	Flexural strength (n=6)		Impact strength (n=4)	
	Median	p-value	Median	p-value
Acralyn normal (short cycle)	52.45	0.015*	61.19	0.200
Acralyn normal (long cycle)	48.10		53.90	
Acralyn Hi-impact (short cycle)	56.95	0.002*	165.20	0.029*
Acralyn Hi-impact (long cycle)	49.15		123.15	

The comparison of the impact strength of Acralyn normal material at different curing cycles revealed that the short curing cycle had a higher impact strength (median: 61.19 J/m) compared to the long cycle (median: 53.90 J/m). However, the difference was not statistically significant (p=0.20). The test results of the impact strength of Acralyn Hi-impact material, which underwent a short cycle, were significantly higher (median: 165.20 J/m) compared to the long cycle (median: 123.15 J/m) (p=0.029) (Table 2).

Comparison of flexural strength between two denture resin materials at short curing cycle exhibited significantly higher values for Acralyn Hi-impact resin (median: 56.95 MPa) than Acralyn normal resin (median: 52.45 MPa) with a p-value of 0.004, whereas the impact strength between the two resins (median Acralyn normal: 48.10 J/m and Acralyn Hi-impact: 49.15 J/m) did not show significant difference in long cycle (p=0.310) (Table 3).

**Table 3:** Comparison of flexural and impact strength between different materials for short and long cycles * significant at p<0.05

Group	Flexural strength (n=6)		Impact strength (n=4)	
	Median	p value	Median	p value
Acralyn normal (short cycle)	52.45	0.004*	61.19	0.029*
Acralyn Hi-impact (short cycle)	56.95		165.20	
Acralyn normal (long cycle)	48.10	0.310	53.90	0.029*
Acralyn Hi-impact (long cycle)	49.15		123.15	

Table 3 shows the comparison of impact strength (median) between two denture resin materials (Acralyn normal: 61.19 and Acralyn Hi-impact: 165.20), showing that there was a significant difference in the short cycle (p=0.029). The long curing cycle also presented significantly higher values for Acralyn Hi-impact resin (median: 123.15) than for Acralyn normal resin (median: 53.90) (p=0.029).

## Discussion

Acrylic dentures fabricated using PMMA are subjected to different types of stresses in the oral cavity. The fracture of the denture bases intraorally is mainly contributed by the fatigue stresses accommodated in the denture while functioning. The extraoral fracture of the denture is mostly caused by the impact stresses on the denture. To withstand these functional and parafunctional masticatory forces and impact forces due to accidental dropping, the acrylic resin needs to have high flexural strength and fracture resistance. The processing technique, chemical composition, and the time and temperature factors of the polymerization techniques can affect the mechanical properties of the denture base resins [11-13].

Among the various polymerization techniques, activation using thermal energy in a hot water bath is a conventionally employed and well-accepted technique. However, the time required for processing is considered a major drawback of this technique [14]. There are various time- and temperature-dependent water bath cycles utilized to process the acrylic resin without losing its desirable properties. The polymerization cycles frequently used for curing the acrylic resins are ideally short or long cycles. So, improvement in physical and mechanical properties along with reduced time is desirable.

Various studies have been conducted regarding the processing techniques and various combinations of acrylic materials [15-18]. Durkan et al. conducted a study on the effect of the polymerization technique on the transverse strength of acrylic resin by comparing the water bath processing technique with autoclave polymerization. It was observed that there was no significant difference between the two polymerization techniques [13].

Many studies have incorporated various reinforcement materials into conventional acrylic compositions, including fibers, metal particles, nanoparticles, and rubber agents [15-17]. Yadav et al. compared the flexural strength of denture base resins reinforced with aluminum oxide and compared two different processing techniques. They concluded that there was no statistically significant difference between the conventional water bath and microwave techniques. Also, aluminum oxide doesn’t show any improvement in the flexural strength of the material [15].

A systematic review and meta-analysis conducted by Somani et al. reported that reinforcement of PMMA can significantly improve the flexural strength and impact strength of the denture base materials [17]. Glass fiber additions have been proven to show improvement in mechanical properties [16]. The rubber-based agents make the denture base resin a high-impact material. Praveen et al. conducted an in vitro study to compare the impact strength and fracture morphology of four types of heat-cured denture base resins [18]. The study reported that the impact strength of the acrylic resin is affected by the reinforcement of fibers. However, only limited studies have been conducted to conclude the difference in impact strength between conventional and high-impact resins.

Charpy's impact test is a mechanical test that determines the impact strength of a material [12]. It is a standardized strain rate test that evaluates the energy absorption happening in a material during fracture. The amount of energy absorbed by the material is calculated as notch toughness. Increasing the impact strength of denture material will improve its function and avoid frequent repairs and replacements. Materials with multiple additions, reinforcement mechanisms, and different processing techniques can impact the strength of acrylic material. Several authors have also utilized the impact test to understand the fracture strength of different acrylic compositions [17-21]. However, it is not exactly possible to compare with the findings of the other studies, mostly due to the difference in study protocol in specimen dimensions, notch geometry, and type of test (Charpy or Izod). Dikbas et al. conducted a study on the impact fracture strength of six different denture materials and processing techniques. Sixty specimens from six groups were analyzed for impact strength. The high-impact strength acrylic material in the water bath polymerization showed the highest mean impact strength, followed by the microwave activation technique and rapid heat polymerization [12]. The three-point bending flexural test is an excellent indicator of the modulus of elasticity while bending and flexural strain in a material [17]. It was conducted in a universal testing machine to measure the flexural strength of acrylic resin. The advantage of the testing technique lies in the ease of specimen preparation and testing method.

In the present study, the null hypothesis was rejected as the impact strength was significantly higher for high-impact acrylic resin (165.20) in comparison with conventional acrylic resin (61.19 J/m) in the short curing cycle. The flexural strength was also superior for high-impact acrylic resin (56.95 MPa) when compared to conventional acrylic resin (52.45 MPa) in the short circle. This was consistent with previous studies, which reported impact strength ranging from 50.9 J/m for the conventional heat-cured resin to 184.31 J/m for the high-impact acrylic material. The flexural strength measurements showed a range of 37.7 MPa in conventional heat-cured resin to 58.1 MPa for high-impact acrylic resin. The impact strength and flexural strength of high-impact acrylic resin processed in a short cycle were found to have the highest value in the present study, and various authors have reported similar results in different settings [17-18]. Dikbas et al. reported an impact strength range of 0.160 J for a light cure material and 0.561 J for a high-impact material [12]. Another study by Craig et al. using the Charpy unnotched technique reported that the reported ranges from 0.26 J for a conventional resin to 0.58 J for a rubber-reinforced denture resin [19]. The lowest values of impact strength and flexural strength were obtained for conventional resin in a long water bath cycle. The results obtained were comparable to those of other similar studies. Seo et al. concluded that the flexural strength of heat-cured resin processed by short water bath cycles was greater compared to long cycles [20]. However, a comparison study conducted by Arundati et al. on an indigenous high-impact acrylic resin with two commercially available conventional acrylic materials reported a higher value of transverse and impact strength for a long water bath cycle compared to a short cycle [21]. The effect of different polymerization cycles of denture base materials can be attributed to the increase in polymerization temperature. Additionally, the residual monomer content decreases due to further polymerization at the active radical sites. At higher temperatures, the monomer molecules are expected to diffuse more quickly to these active sites, resulting in a reduction of the residual monomer content [11].

The study had a few limitations. Mechanical properties like hardness, flexural modulus, and optical properties were not evaluated as they were long-term outcomes. Also, we did not include other reinforced acrylic resins and polymerization techniques and recommend further research in this area.

## Conclusions

High-impact denture base resin provided significantly higher impact strength than conventional resin. The flexural strength was slightly better for high-impact resin than conventional resin. A short curing cycle was more effective and had better mechanical properties than a long curing cycle in the polymerization of both acrylic resins.
